# Structured thoracic computed tomography report for COVID-19 pandemic

**DOI:** 10.31744/einstein_journal/2020ED5720

**Published:** 2020-04-03

**Authors:** Hamilton Shoji, Eduardo Kaiser Ururahy Nunes Fonseca, Gustavo Borges da Silva Teles, Rodrigo Bastos Duarte Passos, Elaine Yanata, Murilo Marques Almeida Silva, Marcelo Buarque de Gusmão Funari, Roberto Sasdelli, Walther Yoshiharu Ishikawa, Rodrigo Caruso Chate, Gilberto Szarf

**Affiliations:** 1 Hospital Israelita Albert Einstein São PauloSP Brazil Hospital Israelita Albert Einstein, São Paulo, SP, Brazil.

By the end of 2019, a novel coronavirus (severe acute respiratory syndrome coronavirus *2* or SARS-CoV-2) was identified as the etiologic agent of an outbreak of pneumonia in the city of Wuhan, province of Hubei, in China. The virus had a rapid dissemination, with person-to-person transmission and cases soon identified throughout the world. The disease was called COVID-19 (coronavirus disease 2019), with several new outbreaks related to community transmission. It is now classified as pandemic.^1 - 4^

We have observed an increase in requests of chest computed tomography (CT) since the first records of cases in Brazil; therefore, in a near future, the current installed capacity of the system to analyze and produce the CT reports may be exceeded. It is crucial to highlight that the definite diagnosis of COVID-19 is made by real-time polymerase chain reaction (RT-PCR), and a normal (negative) chest CT does not rule out diagnosis. However, currently, the RT-PCR result has taken longer than CT reports to be available, so CT has taken an important role in a comprehensive assessment of patients, for demonstrating high sensitivity (although low specificity), to detect the most frequent pulmonary findings of the disease.

In this high demand urgency context to provide results, it is advisable that the content of the radiological report be very objective and as clear as possible for the requesting physicians from the emergency department. The most relevant pieces of information to be conveyed are presence (or not) of pulmonary involvement, if the findings are compatible with infectious process, and, in positive cases, if the changes are suggestive of viral etiology, particularly COVID-19, even if there is overlapping of findings with other infectious diseases (including other viruses). We also included an approximate estimate of extent of pulmonary involvement by the disease (visual analysis), which has been considered useful by them in management of patients, together with other clinical data and physical examination. In our Institution, involvement of >50% of parenchyma^5^ has been used as an additional criterion to decide for hospitalization.

**Figure 1:**
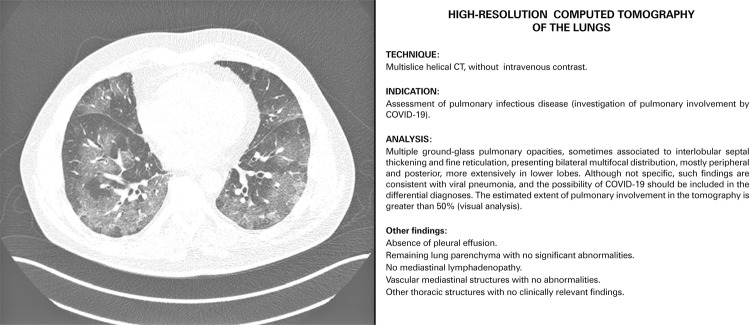
Computed tomography image of a confirmed case of COVID-19 with typical findings and its respective report

In a structured report, we initially describe if there are pulmonary changes or not, and if they are suggestive of a pulmonary infectious process. In case of alterations, if the features are in accordance or not with the more typical pattern described in COVID-19: including ground-glass opacities, sometimes with superimposed interlobular septal thickening (crazy paving), consolidations and reversed halo, presenting a bilateral multilobar distribution, predominantly peripheral, with mild predilection for the posterior regions and lower lobes.^6 - 10^ In these cases, we have highlighted in our reports that “the possibility of COVID-19 should be considered in the differential diagnoses”, and also included the estimated extent of parenchyma involvement (greater or lesser than 50%).^5^Figure 1 shows a case with typical imaging findings of COVID-19 and the model of provided report.

**Figure 2:**
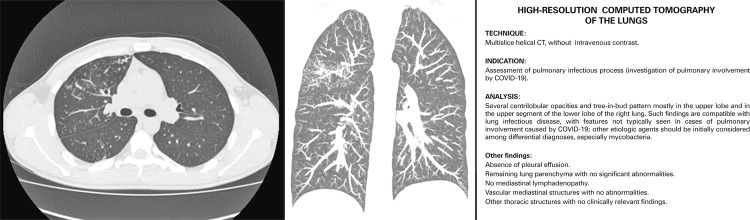
Computed tomography images of a case with initial clinical suspicion of COVID-19 and its respective report, with tomographic findings suggestive of infectious process, but with characteristics that are not usual in COVID-19. After performing ancillary investigations, the diagnosis was pulmonary tuberculosis

In patients with tomographic findings more suggestive of other type of infection which obviously must not be neglected amidst the pandemic, we have described the changes and concluded as follows: “Such findings are compatible with pulmonary infectious process, and its characteristics are not typically observed in cases of pulmonary involvement by COVID-19; other etiologic agents should be initially considered in the differential diagnosis.” As examples of changes described in the literature as uncommon in cases of COVID-19, which increase the probability of infection by other agents, we underline numerous centrilobular micronodules with tree-in-bud pattern, relatively well-defined solid nodules, cavities, predominant central parenchyma involvement, mainly affecting the airways.^6 - 9^Figure 2 displays an example of one of those cases and its respective report, with final diagnosis of tuberculosis after complete investigation.

In the subgroup of patients with no tomographic evidence of pulmonary infectious process, we clearly and explicitly report the following phrase: “Absence of focal pulmonary opacities suggestive of active parenchymal infectious process.”

Next, other additional relevant findings are briefly included, emphasizing the presence or not of lymph node enlargement, pleural effusion, as well as pulmonary nodules, emphysema, chronic interstitial disease, aneurysms and marked atheromatous disease.

Developing structured reports in radiology, primarily those oriented to certain diseases, provides several benefits, including clarity in conveying information to requesting physicians and use of a common terminology, enabling all those involved to be familiar with specific terms used for each disease. Moreover, this later enables collecting data for epidemiological purposes, quality control and research.^11 , 12^ Other advantages comprise increased radiologist productivy with less burden.^12^

Implementing a structured report should be beneficial, particularly in the current scenario of COVID-19 pandemic, for increasing productivity of radiologists and enabling better understanding of the requesting physicians, with a potential positive impact in management of patients.^13^
