# Supplementary material for: Structured thoracic computed tomography report for COVID-19 pandemic
Source: Einstein (Sao Paulo). 2020 Apr 3;18:eED5720. doi: 10.31744/einstein_journal/2020ED5720 (PMC7135926; doi:10.31744/einstein_journal/2020ED5720)
Supplement: Translation

# Translation: Relatório estruturado de tomografia computadorizada de tórax para a pandemia do COVID-19

**Authors:** Hamilton Shoji, Eduardo Kaiser Ururahy Nunes Fonseca, Gustavo Borges da Silva Teles, Rodrigo Bastos Duarte Passos, Elaine Yanata, Murilo Marques Almeida Silva, Marcelo Buarque de Gusmão Funari, Roberto Sasdelli, Walther Yoshiharu Ishikawa, Rodrigo Caruso Chate, Gilberto Szarf

**Affiliations:** 1 Brasil Hospital Israelita Albert Einstein, São Paulo, SP, Brasil.

No final de 2019, um novo coronavírus ( *severe acute respiratory syndrome coronavirus 2* ou SARS-CoV-2) foi identificado como agente etiológico de um surto de pneumonias na cidade de Wuhan, província de Hubei, na China. A disseminação foi rápida, com transmissão de pessoa a pessoa e posterior internacionalização dos casos. A doença passou a ser denominada COVID-19 ( *coronavirus disease 2019* ) e logo atingiu outros continentes, com diversos novos surtos relacionados à transmissão comunitária, sendo agora classificada como pandemia.^1 - 4^

Percebemos um número crescente de solicitações de tomografia computadorizada (TC) de tórax desde os primeiros registros de casos no Brasil, de modo que, em um futuro próximo, é possível que a capacidade do sistema atualmente instalado para análise e confecção de relatórios tomográficos seja ultrapassada. É fundamental pontuar que o diagnóstico definitivo de COVID-19 é realizado pela reação em cadeia da polimerase em tempo real (RT-PCR) e a TC de tórax normal (negativa) não o exclui. Porém, como o resultado da RT-PCR, pelo menos por ora, tem sido disponibilizado dentro de um prazo mais longo que o relatório da TC, esta passou a assumir papel importante dentro da avaliação global dos pacientes, inclusive por já ter se mostrado bastante sensível, ainda que pouco específica, na detecção dos achados pulmonares mais frequentes na doença.

Neste contexto de alta demanda e urgência para disponibilização dos resultados, é desejável que o conteúdo do relatório radiológico seja bastante direto e o mais claro possível para os médicos solicitantes do setor de emergência, também bastante sobrecarregados nesse cenário de pandemia. A informação mais relevante a ser passada diz respeito à presença (ou não) de acometimento pulmonar, se o aspecto dos achados é compatível com processo infeccioso e, nos casos positivos, se as alterações sugerem etiologia viral, particularmente de COVID-19, mesmo que exista sobreposição dos achados com outras doenças infecciosas (incluindo outros vírus). Inserimos também uma estimativa aproximada da extensão do acometimento pulmonar pela doença (análise visual), o que tem sido considerado útil por eles no manejo dos pacientes, juntamente de outros dados clínicos e exame físico – em nosso serviço, o envolvimento de >50% do parênquima^5^ tem sido utilizado como critério adicional para a decisão de internação.

De forma estruturada, inicialmente descrevemos se há ou não alterações pulmonares cujo aspecto seja sugestivo de processo infeccioso pulmonar. Quando presentes, se as características seguem ou não o padrão mais frequentemente descrito na COVID-19, incluindo opacidades em vidro fosco, pavimentação em mosaico, consolidações e halo invertido, apresentando distribuição geralmente bilateral e multilobar, predominantemente periférica, com leve predileção pelas regiões posteriores e pelos lobos inferiores.^6 - 10^ Nos casos que reúnem essas características, temos destacado, em nossos relatórios, que “a possibilidade de COVID-19 deve ser considerada entre os diagnósticos diferenciais”, incluindo, ainda, a estimativa da extensão do envolvimento parenquimatoso (superior ou inferior a 50%).^5^ A figura 1 mostra um caso com características compatíveis com COVID-19 e o modelo de relatório disponibilizado.

**Figura 1:** Imagem tomográfica de caso confirmado de COVID-19 com achados típicos e seu respectivo relatório

Nos pacientes com achados tomográficos mais sugestivos de outro tipo de infecção e que, naturalmente, não devem ser negligenciados, mesmo em meio à pandemia, temos procurado descrever as alterações e concluir da seguinte forma: “Tais achados são compatíveis com processo infeccioso pulmonar, cujas características não são tipicamente observadas nos casos de comprometimento pulmonar por COVID-19; outros agentes etiológicos devem ser inicialmente considerados entre os diagnósticos diferenciais”. Como exemplos de alterações descritas na literatura como incomuns nos casos de COVID-19 e que aumentam a probabilidade de infecção por outros agentes, destacamos numerosos micronódulos centrolobulares com padrão de árvore em brotamento, nódulos sólidos relativamente bem definidos, cavidades, predomínio de acometimento central do parênquima e envolvimento preferencialmente de vias aéreas.^6 - 9^ A figura 2 mostra um exemplo desses casos e seu respectivo relatório, com diagnóstico final de tuberculose após a investigação completa.

**Figura 2:** Imagens tomográficas de caso com suspeita clínica inicial de COVID-19 e seu respectivo relatório, com achados tomográficos sugestivos de processo infeccioso, cujas características, porém, não são usuais na COVID-19. Após a investigação complementar finalizada, o diagnóstico foi de tuberculose pulmonar

No subgrupo de pacientes sem evidências tomográficas de processo infeccioso pulmonar, temos deixado essa informação de forma clara e explícita no relatório, utilizando a seguinte frase: “Ausência de opacidades pulmonares focais sugestivas de processo infeccioso parenquimatoso em atividade.”

Outros achados adicionais relevantes são incluídos de forma sucinta na sequência, destacando-se a presença ou não de linfonodomegalias, existência ou não de derrame pleural, bem como achados como nódulos pulmonares, enfisema, intersticiopatia crônica, aneurismas e ateromatose significativa.

A confecção de relatórios estruturados em radiologia, principalmente aqueles dirigidos a determinadas doenças, oferece diversos benefícios, entre os quais se destacam a clareza com que as informações são transmitidas aos médicos solicitantes e a utilização de um léxico comum, o que facilita a familiarização de todos os envolvidos com os termos específicos utilizados em cada doença, além de, posteriormente, facilitar a coleta de informações para fins epidemiológicos, controle de qualidade e pesquisa.^11 , 12^ Também são citados como vantagens a maior agilidade na elaboração dos relatórios e um menor desgaste por parte dos radiologistas.^12^

A implementação de um relatório estruturado deve ser benéfica, particularmente no cenário atual de pandemia por COVID-19, por melhorar a produtividade do radiologista e facilitar o entendimento das informações por parte do médico solicitante, com potencial impacto positivo na conduta frente ao paciente.^13^
